# Associations and interactions between variants in selenoprotein genes, selenoprotein levels and the development of abdominal aortic aneurysm, peripheral arterial disease, and heart failure

**DOI:** 10.1371/journal.pone.0203350

**Published:** 2018-09-06

**Authors:** Ewa Strauss, Jolanta Tomczak, Ryszard Staniszewski, Grzegorz Oszkinis

**Affiliations:** 1 Institute of Human Genetics, Polish Academy of Sciences, Poznan, Poland; 2 Department of General and Vascular Surgery, Poznan University of Medical Sciences, Poznan, Poland; 3 Department of Vascular and Endovascular Surgery, Angiology and Phlebology, Poznan University of Medical Sciences, Poznan, Poland; Robert Gordon University, UNITED KINGDOM

## Abstract

**Background:**

Little is known on the role of selenoprotein genes in cardiovascular disease. This study examines the associations of the *SEPP1*, *SELENOS*, *TXNRD1*, *TXNRD2*, *GPX4*, and *SOD2* polymorphisms and selenoprotein P (SeP) and thioredoxin concentrations with the development of abdominal aortic aneurysm (AAA) and aortoiliac occlusive disease (AOID), as well as their influence on cardiac phenotype.

**Methods:**

564 patients with AAA, 400 patients with AIOD, and 543 controls were enrolled and characterized for coronary artery disease, myocardial infarction, and systolic heart failure (HF) occurrence. In AAA, the coexistence of peripheral arterial disease (PAD) was examined. Genotypes were determined using TaqMan-based assays. Selenoprotein concentration was assessed using the ELISA method.

**Results:**

The *SELENOS rs34713741T*, *SEPP1 rs3877899A*, and *GPX4 rs713041T* alleles were related to a 30–60% increase in the AIOD/PAD risk in the recessive or dominant model (all associations at *P* < .05). The *SEPP1 rs3877899A* allele was a protective factor for the development of AAA without concomitant PAD (OR = 0.68 for the dominant model, *P* = .014), but not AAA with concomitant PAD. The cumulative two-locus effects of selenoprotein genes on the AAA/AIOD risk were observed, including the multiplicative interaction between the *SELENOS rs34713741T* and *GPX4 rs713041T* alleles (both in the recessive model) affecting the AIOD risk (OR = 5.27, *P* = .001) and its clinical phenotype. Coexistence of HF in aortic diseases was related to both the *SEPP1 rs7579A* allele (OR = 1.83 for carriers, *P* = .013) and increased SeP concentrations; SeP level ≥8.5 mg/mL caused a 3.5-fold increase in the risk of HF. In AAA, SeP levels were correlated with BMI (*r* = -0.575, *P* < .0001).

**Conclusions:**

Our results provide evidence that selenoprotein polymorphisms constitute a risk factor for HF and peripheral atherosclerosis, but prevent the development of AAA. Excessive weight might result in reduced antioxidant reserve efficiency in AAA. Validation studies are required to establish whether SeP concentration may be a marker for HF.

## 1. Introduction

Enhanced oxidative stress in cardiac and vascular myocytes contributes to the pathogenesis of cardiovascular disease (CVD) [1]. Selenium (Se), as a micronutrient with antioxidant activities, protects tissues from oxidative damage and thus exerts beneficial effects on the cardiovascular (CV) system [2]. Endemic deficiency of Se causes Keshan (cardiomyopathy) or Kashin-Beck (osteoarthritis) diseases [3], while suboptimal Se status of the body has been linked to the development of cancer and CVD, including heart failure (HF) [4, 5], abdominal aortic aneurysm (AAA) [6], and Buerger disease [7], as well as to increased all-cause and CV mortality in elderly populations [8]. Se status in Europeans, particularly those inhabiting the central part of the continent, is below recommended values [8–10]. This is a result of low soil Se content and lack of its supplementation in food products [2].

Se exerts its biological role as a component of 25 selenoproteins, into which it is incorporated in the form of the amino acid selenocysteine [11]. These proteins are involved in the cellular antioxidant defense system and have anti-inflammatory activities. They comprise selenoprotein P (SeP), which is the main selenoprotein in plasma with both Se transport and antioxidant activities, the thioredoxin reductases (TrxRs), which control the intracellular redox environment, selenoprotein S (SelS), which is involved in the inhibition of endoplasmic reticulum stress, and glutathione peroxidase 4 (PHGPx), an enzyme active in the protection of cells from membrane lipid peroxidation.

Several single-nucleotide polymorphisms (SNPs) in selenoprotein genes have been shown to have functional consequences. They influence gene expression [12–14] or protein function, and eventually the selenoprotein status *in vivo* [15, 16]. Many previous studies revealed strong associations between variants in selenoprotein genes and malignant diseases [17]. They pointed out, that these relationships may be partially a consequence of interactions between selenoprotein gene variants and variants in other genes that encode for proteins involved in antioxidant defense. Among them, interactions with the manganese superoxide dismutase gene (*SOD2*) variants are observed the most frequently [18–20]. There is also growing evidence on the role of polymorphisms in selenoprotein genes in CVDs [21–29].

AAA and aortoiliac occlusive disease (AIOD) are two CVDs which partially share their location and risk factors. AAA is characterized by a progressive dilatation of the infrarenal abdominal aorta, with the diameter exceeding 3 cm. The disease is frequently asymptomatic until the rupture of the aorta. The mechanisms of aneurysm initiation and rupture are still not fully elucidated, partly because of the coexistence of atherosclerosis in the majority of cases. This hinders the designation of specific risk factors. AIOD is a subtype of peripheral arterial disease (PAD), in which the atherosclerotic occlusions are located prevalently in the aorta and the iliac arteries. The disease leads to ischemia of the lower extremities. If untreated, it can result in the loss of the lower limbs. Both AAA [30] and PAD [31] are related to premature CV mortality not only in consequence of disease progression, but also as a result of sudden CV event or the development of chronic heart failure (HF). The hallmarks of AAA and PAD pathogenesis include local inflammation and oxidative stress. Tobacco smoke exposure and the aging process contribute to reduced antioxidant reserve in these diseases through mechanisms that include excessive utilization of Se [8, 32].

Considering the crucial role of oxidative stress in the development of AAA and PAD, it would be plausible to hypothesize that SNPs in genes modulating the defense system against oxidative stress will also affect the risk and course of these diseases. This approach, known as Mendelian randomization [33], may strengthen the ability of case-control studies to draw causal inferences. Accordingly, we clarify the associations of SNPs in the *SEPP1*, *SELENOS*, *TXNRD1*, *TXNRD2*, *GPX4*, and *SOD2* genes, and the selenoprotein P (SeP) and thioredoxin (Trx) concentrations with the development of AAA and AIOD/PAD, as well as their influence on cardiac phenotype (history of coronary artery disease (CAD), myocardial infarction (MI), and systolic HF) in these diseases. Except for our preliminary results on the *SEPP1* SNPs [29], there is no previously published work on the distribution of selenoprotein SNPs in aortic diseases. These SNPs were not analyzed in HF.

## 2. Materials and methods

### 2.1. Study population

The study population comprised 564 patients with AAA (24.1% of them with small AAA <5.5 cm and 34.6% with AAA >6.6 cm at the time of sample collection) and 400 patients with AIOD scheduled for elective surgery at the Department of General and Vascular Surgery of the Poznan University of Medical Sciences in the years 1999–2011. The diagnosis of AAA was evaluated by computed tomography angiography or magnetic resonance angiography, while the diagnosis of AIOD–by computed tomography angiography alone. The control group, consisting of 543 subjects older than 45 years with no evidence of AAA or PAD, was selected at the same time from the Poznan district. Clinical data were collected, including: demographic parameters (age and gender), risk factor distribution (cigarette smoking history, arterial hypertension (AH), diabetes, obesity, blood lipid and lipoprotein profiles), and the presence of comorbidities: PAD (examined in 92.6% of AAA cases), CAD, MI, and systolic HF (defined as left ventricular ejection fraction below or equal to 40%).

Plasma samples were available from patients collected in the years 2010–2011. To investigate the role of the concentration of selenoproteins a group of 70 AAA patients (aneurysms ≥ 5.5 cm, 50% with coexisting PAD), 40 AIOD patients, and 40 controls was selected (subsequent cases and controls matched by age and sex). Based on preliminary results, patients with coexisting HF were excluded from further analysis of AAA and AIOD (5 AAA patients and 3 AIOD patients), and all AAA patients were analyzed together, regardless of the coexistence of PAD. The rest of group of patients with coexisting HF (N = 15) was chosen from the stored plasma samples (all available samples from patients with HF were examined).

All experiments were carried out in compliance with the relevant laws and guidelines in accordance with the ethical standards of the Declaration of Helsinki. The study protocol was approved by the Bioethical Committee of the Poznan University of Medical Sciences, Poland (decision nos. 628/10, 796/15, and 797/2015), and all subjects provided their informed written consent to participate in the study.

### 2.2. Biological samples collection

Blood samples were collected using ethylenediaminetetraacetic acid as an anticoagulant. For plasma preparations, 9 ml samples of peripheral blood were acquired from an antecubital vein and centrifuged for 15 minutes at 1000 x g (8°C) within 30 minutes from collection. The plasma samples were aliquotted and stored at—80°C. After thawing, they were centrifuged again before the assay. The remaining part of each sample, comprising blood lymphocytes, was used for genomic DNA isolation. DNA was extracted by a chemical method, using the acid guanidinium thiocyanate-phenol-chloroform extraction.

### 2.3. Biochemical analyses and genotyping

The plasma concentrations of SeP and Trx were measured with the ELISA method using commercially available tests (Antibodies Online, Cusabio Biotech Co; test numbers: CSB-EL021018HU and CSB-E09728h, respectively). For SeP, 5 ml samples of plasma were diluted in accordance with the protocol and incubated with reagents. The plasma concentrations of SeP and Trx were calculated based on the standard curve. Seven SNPs (*SEPP1 rs3877899* and *rs7579*, *SELENOS rs34713741*, *TXNRD1 rs35009941*, *TXNRD2 rs9605031*, *GPX4 rs713041*, and *SOD2 rs4880*) were assessed using predesigned TaqMan SNP Genotyping Assays and the ABI 7900HT Fast Real-Time PCR System (Life Technologies, Carlsbad, California). The details of the allele discrimination tests and location of studied SNPs are available in S1 Table. The genotyping success rate was 99%. Samples with failed genotype calls were excluded from the analysis.

### 2.4. Statistical analysis

Genotype frequencies were tested for the Hardy-Weinberg equilibrium (HWE) using a *χ*^*2*^ test (http://ihg.gsf.de/cgi-bin/hw/hwa1.pl). Haploview was used to determine linkage disequilibrium (LD) between the *SEPP1* variants and to estimate haplotype frequencies. Univariate analyses were performed, in which a *t-*test was used for quantitative variables, and the *χ*^*2*^ test was employed for qualitative variables. Continuous variables with non-normal distribution were analyzed after logarithmic transformation. Alternatively, non-parametric tests were used: Mann-Whitney U test and Kruskal-Wallis ANOVA (comparison of the selenoprotein concentration between the studied groups). Pearson's coefficient of correlation (*r*) was calculated to assess the strength of the observed correlations. For genotypes, alleles, haplotypes, and other qualitative variables, the values of odds ratios (ORs) and 95% confidence intervals (95% CI) were calculated. The two-locus interactions between studied variants were evaluated using the four-by-two tables. The synergic effect of the genotypes was recognized when observed OR was higher (or lower), than OR expected from an additive model of interaction: OR_SNP1xSNP2_ (OR_*Observed*_) > or <OR_SNP1_ + ORSNP2−1 (OR_*Expected*_). Models for younger and older subgroups were considered. Multivariate regression analysis was performed to adjust the impact of genotype for known CV risk factors. The observed differences were considered significant at *P* < .05. All analyses were performed using STATISTICA software version 10.0 and GraphPad Prism version 6.04.

## 3. Results

### 3.1. Traditional risk factor distribution and concomitant diseases

Smoking was the most prevalent and the most important risk factor for AAA and AIOD (respectively, 81.9% and 91.0%, as compared to 37.4% in controls, *P* < .0001; Table 1). The other risk factors for both aortic diseases were AH and decreased high-density lipoprotein cholesterol (HDLC) level. Obesity was relatively rare in studied aortic diseases, in particular in AIOD (13.8% vs 25.8 in controls; *P <* .0001). There were no differences between cases and controls regarding the frequency of coronary artery disease (CAD; 34.1–49.8%), whereas a history of myocardial infarction (MI) was more often found in AAA (31.4%) and AIOD (25.8%), than in controls (15.8%; *P* < .01 and *P* < .05, respectively). The frequency of systolic HF was higher in AIOD (10.3%) than in AAA (7.1%, *P* = .006). In 50.5% of AAA patients, the coexistence of PAD was observed, which was associated with higher incidence of AH (80.7% vs. 68.4%, *P* = .006; S2 Table). Comparing CV risk factors between groups of patients we found, that advanced age, male gender, AH, and obesity constituted risk factors that were more common in AAA, while diabetes and smoking were risk factors of higher frequency in the AIOD (Table 1).

**Table 1:** Demographic and clinical characteristics of patients with abdominal aortic aneurysm (AAA), patients with aortoiliac occlusive disease (AIOD), and control subjects.

Variable	Controls N = 543	AAA N = 564	AIOD N = 400	*P* (if significant)		
				AAA vs Controls	AIOD vs Controls	AAA vs AIOD
Median age [years], Q_25_-Q_75_	62 (56–69)	68 (61–74)	60 (54–67)	< .0001		< .0001
Age range [years]	45–86	45–94	43–86			
Male sex, n (%)	391 (72.0)	487 (86.3)	288 (72.0)	< .0001		< .0001
Smoking (past or present), n (%)	203 (37.4)	462 (81.9)	364 (91.0)	< .0001	< .0001	< .001
Arterial hypertension, n (%)	232 (42.7)	416 (73.8)	264 (66.0)	< .0001	< .0001	< .05
Diabetes, n (%)	77 (14.2)	92 (16.3)	93 (23.3)		< .01	< .001
Obesity (BMI ≥ 30 kg/m^2^), n (%)	140 (25.8)	107 (19.0)	55 (13.8)		< .0001	< .01
Lipid and lipoprotein profile [mmol/L], Q_25_-Q_75_						
TC	5.61 (4.71–6.54)	5.09 (4.26–6.03)	5.27 (4.38–6.38)		< .05	
HDLC	1.42 (1.11–1.69)	1.11 (0.92–1.36)	1.15 (0.95–1.40)	< .0001	< .0001	
LDLC	3.45 (2.96–4.33)	3.10 (2.30–4.00)	3.20 (2.31–4.20)			
TG	1.41 (1.06–2.00)	1.43 (1.05–2.02)	1.57 (1.12–2.17)			
Hyperlipidemia, n (%)	318 (58.6)	341 (60.5)	254 (63.5)			
Comorbidities, n (%)						
Coronary artery disease	185 (34.1)	281 (49.8)	169 (42.3)			
Myocardial infarction	86 (15.8)	177 (31.4)	103 (25.8)	< .05	< .01	
Systolic heart failure	0 (0.0)	40 (7.1)	41 (10.3)			< .01
Peripheral arterial disease	0 (0.0)	263 (50.4)	400 (100.0)			< .0001

*BMI*, body mass index; *HDLC*, high-density lipoprotein cholesterol; *LDLC*, low-density lipoprotein cholesterol; *TC*, total plasma cholesterol; *TG*, triglyceride.

Variables are expressed as median (interquartile range, 25th and 75th percentiles: Q25–Q75) or as numbers (percentages). The differences in the distribution of potentially modifiable vascular risk factors were evaluated with adjustment for age (in 10-year strata) and sex, using logistic or conditional regression analysis.

### 3.2. Genotype distribution

Except for the frequency of the *SELENOS rs34713741* genotypes in the control group (*P* = .028), the distribution of genotypes did not deviate from the Hardy-Weinberg equilibrium (HWE) in either the patients or the controls (S2 Table). The *SEPP1* SNPs were in complete LD, and only three from six possible *rs3877899-rs7579* haplotypes were found (*G-G*, *G-A*, and *A-G*). As a consequence, the effects of the *rs3877899A* and *rs7579A* alleles correspond to the impacts of the *A-G* and *G-A* haplotypes, respectively. The low frequency of the studied *TXNRD1* polymorphism (<1%) indicates a mutation rather than a common variant, and was rejected from further analysis.

### 3.3. Effects of single alleles

#### 3.3.1. AAA and AIOD/PAD risk

**Fig 1:**
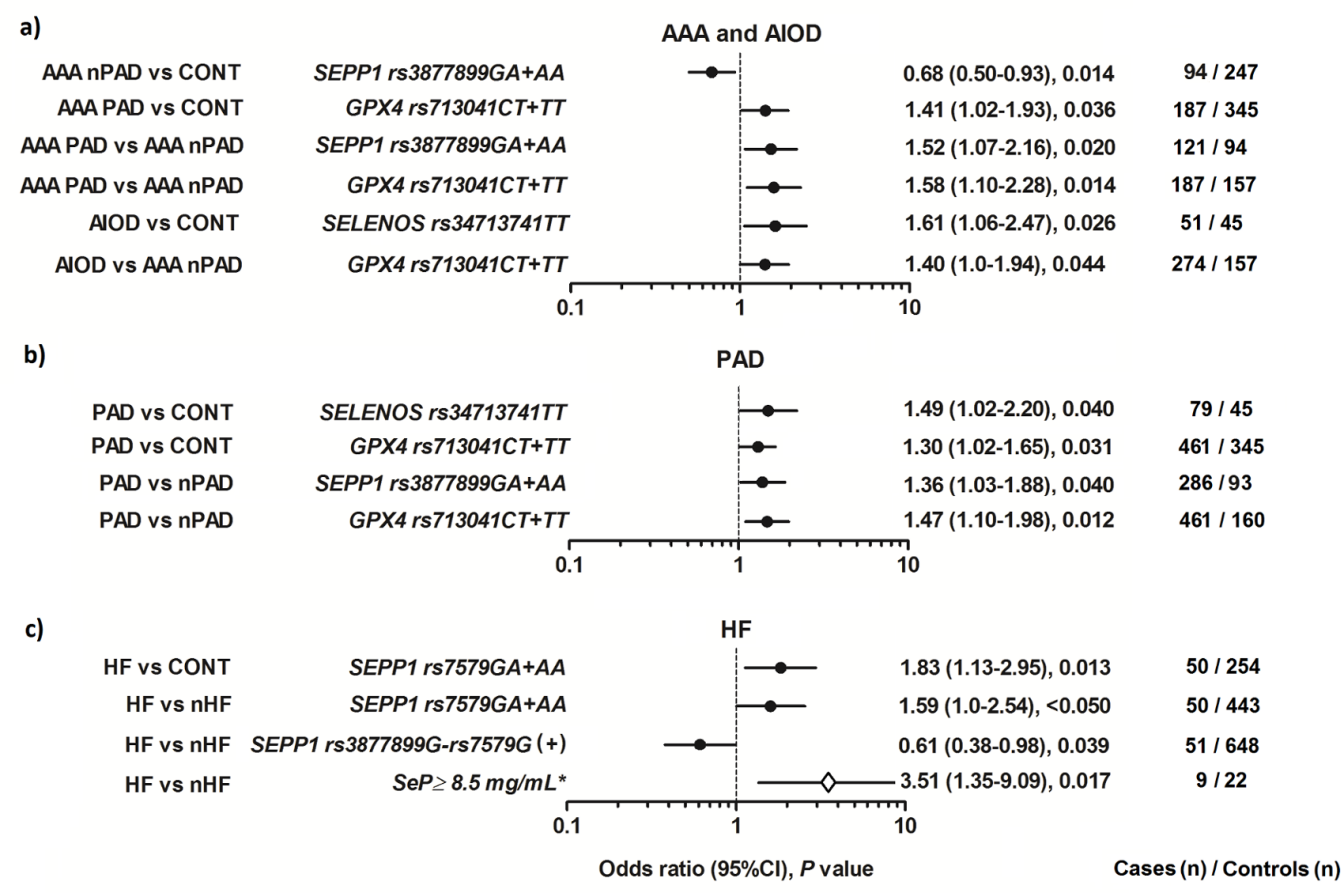
Significant associations between the studied SNPs and SeP levels, and (a) development of the studied aortic diseases: abdominal aortic aneurysm (AAA; N = 564) or aortoiliac occlusive disease (AIOD; N = 400), and (b) occurrence of systolic heart failure (HF; N = 81) and peripheral arterial disease (PAD; N = 664) in the studied patients. Effects in relation to the control group (CONT; N = 543) or corresponding reference group: without PAD (nPAD; N = 259) or without HF (nHF; N = 883) are shown. In evaluating the significance of SeP levels (*), all individuals with a known concentration of SeP were included: 23 patients with HF and 142 subjects without HF.

Results of the analysis of association are presented in Fig 1. Detailed distribution of genotypes and genotype data are shown in S3–S5 Tables.

In this study, the *SEPP1 rs3877899A*, *GPX4 rs713041T* and *SELENOS rs34713741T* alleles were associated with the presence of PAD/AIOD in dominant (*SEPP1* and *GPX4)* or recessive (*SELENOS)* models. These alleles were related to a 40–61% higher probability of occurrence of PAD/AIOD in the analyzes, which compared the frequency of genotypes between the subgroup of AAA patients with PAD and the control group (*GPX4*: OR = 1.41), the subgroup of AAA patients with PAD and the subgroup of AAA patients without PAD (*SEPP1*: OR = 1.52, *GPX4*: OR = 1.58), the AIOD patients and the control group (*SELENOS*: OR = 1.61), and the AIOD patients and the subgroup of AAA patients without PAD (*GPX4*: OR = 1.4; Fig 1A and S3 Table). On the other hand, the *SEPP1 rs3877899A* allele was related to lower probability of the development of AAA without PAD (vs. controls: OR = 0.68).

The association of studied *SEPP1*, *GPX4* and *SELENOS* SNPs with PAD was also confirmed in the evaluation of the whole group of subjects diagnosed with PAD (in the group, which was a combination of AIOD patients and patients with AAA and concomitant PAD). The respective ORs for studied variants, observed in the case-control analysis were: *SELENOS* = 1.49 and *GPX4 =* 1.30, while observed in the analysis that compares genotype frequency between subgroups of studied patients classified for the occurrence of PAD were: *SEPP1 =* 1.36 and *GPX4 =* 1.47 (Fig 1B and S4 Table).

All described above associations were significant at *P* < .05 or *P* = .01.

Because the patients and controls differed in the frequency of MI, all associations observed in the case-control analysis were re-evaluated using controls with or without concomitant MI. We found that the coexistence of MI does not change the direction of the originally observed associations. However, in the case of the *SEPP1* and *SELENOS* SNPs, the associations were more significant after excluding MI, while in the case of the *GPX4* SNP–in the analysis that includes controls with MI.

#### 3.3.2. The cardiac phenotype in aortic disease

From studied concomitant heart diseases (CAD, MI, and HF), only presence of HF showed a relationship with the selenoprotein genotype. The *SEPP1 rs7579A* allele was associated with the presence of HF in both: the case-control analysis (OR = 1.83, *P* = .013), and in the analysis that compared the frequency of genotypes between subgroups of patients classified for the occurrence of HF (OR = 1.59, *P* < .05). On the other hand, the presence of the *SEPP1 rs3877899G*-*rs7579G* haplotype was related to a lower probability of coexistence of HF in studied aortic diseases (OR = 0.61, *P* = .039; Fig 1C and S4 Table).

Similarly to the patients, there was no relationship between the studied variants and CAD or MI in the control group. Furthermore, the coexistence of MI in controls did not affect the association between the *SEPP1 rs7579A* allele and HF.

### 3.4. Cumulative effects of SNPs and gene-gene interactions

The impact of selenoprotein genotypes on the risk of aortic diseases was stronger and substantially more significant, when two-locus effects were considered (S5 Table), which suggested the cumulative effect of allelic variation. For example, the risk of both aortic diseases was influenced by the *GPX4 rs713041CC/ SEPP1 rs3877899GA+AA* genotype (OR = 0.55, 95%CI (0.41–0.73), *P <* .0001) and the *TXNRD2 rs9605031TT*/*SELENOS rs34713741TT* genotype (OR = 15.4, 95%CI (0.91–256.9), *P* = .006). Other observed effects were specific for the type of studied disease. The risk of AAA was influenced by specific two-locus effects between the *SEPP1*, *SELENOS*, and *TXNRD2* genes, which resulted in 50% reduced susceptibility to aneurysm development. The risk of AIOD was influenced by two-locus effects which involve all of the studied selenoprotein genes, and resulted in increased susceptibility to AIOD development with ORs between 1.59 and 6.00.

Observed two-locus effects were analyzed in search of an interaction between studied genes using the four-by-two tables. Among the evaluated associations, only the influence of the *GPX4* and *SELENOS* genes on the risk of AIOD indicated a multiplicative interaction. The presence of the *GPX4rs713041TT/SELENOS rs34713741TT* double homozygous genotype, compared to the occurrence of the *rs713041CC+CT/rs34713741CC+CT* genotype was related to 5.27-fold increase in the risk of AIOD in the case-control analysis (*P* = .0001), and 3.15-fold increase in the risk of this disease in relation to the risk of AAA (*P* = .009; Table 2). The corresponding OR values, estimated on the basis of the sum of the effects of a single genotype (additive effects), were lower and equaled: 1.18 and 1.17. This interaction between *GPX4* and *SELENOS* genes became stronger in older age groups, and in those over 61 years of age, the observed ORs were 7.96 (*P* = .003) and 4.81 (*P* = .002), respectively. Interestingly, analysis of phenotype of carriers of the *GPX4/SELENOS* double homozygous genotype in AIOD in relation to phenotype of carriers of other genotype combinations, showed not only a positive correlation with the patient’s age (65.47 ± 5.74 versus 60.56 ± 8.67, *P* = .031), but also associations with a higher incidence of diabetes (7.7% versus 2.7%, *P* = .029) and obesity (9.8% versus 3.1%, *P* = .025).

**Table 2:** Two-locus interactions between the *SELENOS* and *GPX4* genes in relation to the risk of aortoiliac occlusive disease (AIOD).

Genotype		Controls N = 543	AAA N = 564	AIOD N = 400	OR_*Obs*_ (95%CI), *P*	
*SELENOS* *rs34713741*	*GPX4* *rs713041*				AIOD vs Controls	AIOD vs AAA
All subjects						
*CC+CT*	*CC+CT*	405	423	288	1.0 (reference)	1.0 (reference)
*CC+CT*	*TT*	41	55	36	1.24 (0.77–1.98), .381	0.96 (0.62–1.50), .863
*TT*	*CC+CT*	91	78	61	0.94 (0.66–1.35), .746	1.15 (0.80–1.66), .459
*TT*	*TT*	4	7	15	**5.27 (1.73–16.06), .001**	**3.15 (1.27–7.82), .009**
					OR_*Exp*_ = 1.18 < OR_*Obs*_	OR_*Exp*_ = 1.11 < OR_*Obs*_
Subjects aged > 61 years						
*CC+CT*	*CC+CT*	181	328	125	1.0 (reference)	1.0 (reference)
*CC+CT*	*TT*	34	64	25	1.07 (0.61–1.87), .885	1.03 (0.62–1.70), .898
*TT*	*CC+CT*	17	40	13	1.11 (0.52–2.36), .847	0.85 (0.44–1.65), .745
*TT*	*TT*	2	6	11	**7.96 (1.74–36.6), .003**	**4.81 (1.74–13.3), .002**
					OR_*Exp*_ = 1.17 < OR_*Obs*_	OR_*Exp*_ = 0.88 < OR_*Obs*_

*AAA*, abdominal aortic aneurysm; *OR*_*Exp*_, odds ratio expected from additive model; *OR*_*Obs*_, odds ratio observed

### 3.5. Multivariable analysis

We used logistic regression to examine whether the associations of studied SNPs with AAA and AIOD are significant after adjustment for the traditional CV risk factors (S6 Table). Six different models have been developed, in which the influence of age, sex, and cardiometabolic risk factors (obesity, diabetes and decreased HDLC levels) were evaluated. Smoking and AH were excluded from these analyses as they were present in the majority of studied patients. The association of the *SELENOS rs34713741C>T*, *GPX4 rs713041C>T*, *SEPP1 rs3877899G>A*, and *TXNRD2 rs9605031C>T* SNPs with studied CVD remained significant after adjustment for other risk factors in the analysis of individual or combined genotypes. The *SEPP1 GA+AA/ GPX4 CC* genotype was an independent protective factor for both aortic diseases (OR = 0.46, *P* < .0001), while the *SELENOS TT* (OR = 1.91, *P* = .009), *SELENOS TT/GPX4TT* (OR = 5.43, *P* = .005), *GPX4 CT+TT/TXNRD2 CT+TT* (OR = 1.74, *P* = .009), and *SELENOS TT/SEPP1 GG+GA* (OR = 2.27, *P* = .002) were the most important predictors of AIOD. The association of the *GPX4CT+TT* genotypes with AAA with concomitant PAD (OR = 1.74, *P* = .005) and the *SELENOS TT/ GPX4 TT* genotype with the development of AIOD versus the development of AAA (OR = 4.85, *P* = .002) were also confirmed in multivariable analysis.

### 3.6. Selenoprotein concentration in the studied diseases

**Fig 2:**
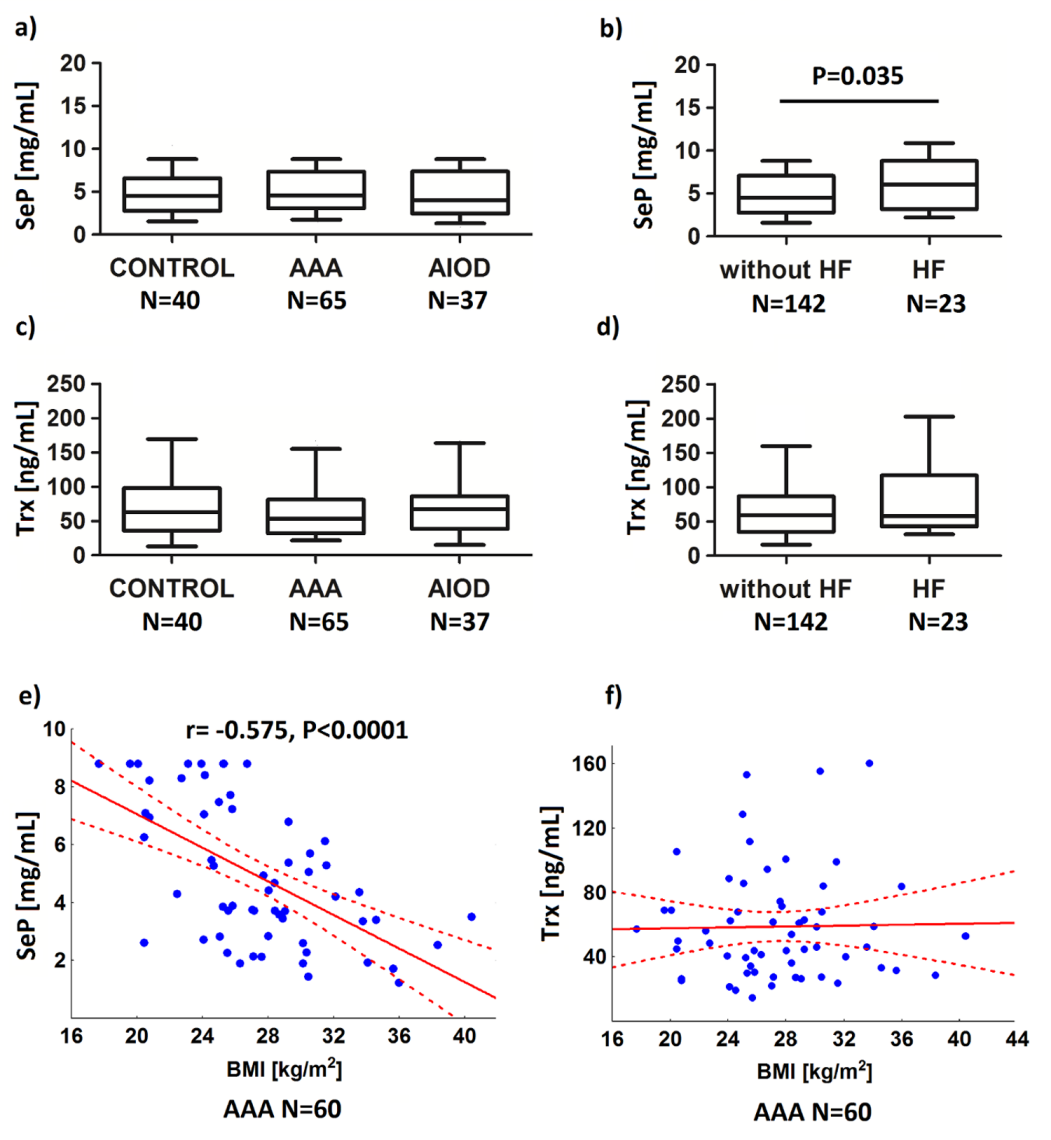
Relationships between plasma selenoprotein P (SeP) and thioredoxin (Trx) concentration and studied diseases (a-d), and BMI in the abdominal aortic aneurysm (AAA; e-f). Patients with AAA, patients with aortoiliac occlusive disease (AIOD) and the control group (CONTROL), as well as the AAA/AIOD patients classified into subgroups according to the presence of heart failure (HF) were analyzed.

We observed significantly increased plasma levels of SeP in patients with concomitant HF, as compared to levels in patients without HF (respectively: 6.17±2.78, N = 23 and 4.94±2.49 mg/ml, N = 142, *P* = .035; Fig 2B). In an analysis in which all subjects with a known concentration of SeP were included (N = 165), the presence of SeP levels ≥ 8.5 mg/ml was associated with a 3.51-fold increased risk of HF (95%CI: 1.35–9.09; *P* = .017; Fig 1C). Increased levels of SeP were found in 9 out of 23 subjects with HF (39.1%) and 22 out of 142 subjects without HF (15.5%). Moreover, in HF, SeP levels were correlated with the dose of the *SEPP1 rs3877899G*-*rs7579G* haplotype (*r =* - 0.467, *P* = .025, N = 23). The levels of Trx were also higher in HF, but not significantly (67.2±41.5, N = 23 and 76.5±48.4 ng/ml, N = 142, *P* = .332, Fig 2D). There were no differences in the concentration of SeP and Trx between AAA (N = 40), AIOD (N = 65), and controls (N = 37; Figs 2A and 2C), also after exclusion of patients with HF from the analysis. The association with PAD was also not found.

The correlations between selenoprotein levels and genotypes were studied. It was found, that the *SELENOS rs34713741T* allele was correlated with the SeP concentration in AIOD (*r* = .443, *P* = .006, N = 37) and the Trx levels in AAA (*r* = .283, *P* = .023, N = 64), while the *SEPP1 rs3877899A* was correlated with the Trx levels in controls (*r* = .346, *P* = .029, N = 40). However, in the analysis of the whole population, there was no correlation between the studied polymorphisms (allelic dosage model) and selenoprotein levels.

Finally, the correlations between selenoprotein levels and BMI values were analyzed, because in our previous study we found that the *SEPP1* haplotype is a risk factor for AAA in overweight and obese subjects [29]. In AAA a negative correlation between the concentration of SeP and BMI was observed (*r* = -.575, *P <* .0001, N = 60; Fig 2E). The level of Trx was associated with BMI neither in AAA (*r* = —.019, *P =* .885, N = 60; Fig 2F), nor in other studied groups.

## 4. Discussion

**Fig 3:**
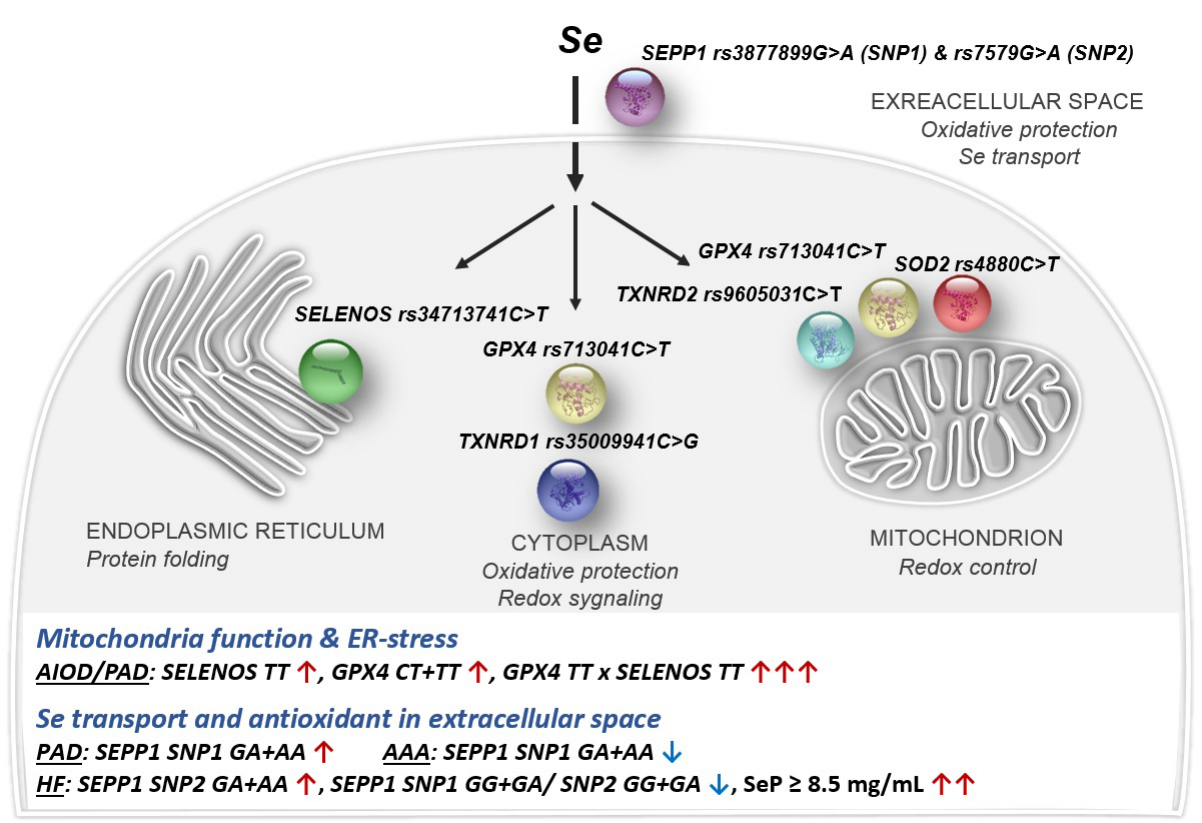
Selenoprotein subcellular localization, biological function and the major findings of this study. Black and blue arrows indicate the strength and direction of influence.

The results of the presented study provide preliminary evidence of the role of SeP, and functional SNP in the gene encoding this protein in systolic HF, and showed complex relationships between variation in selenoprotein genes and pathogenesis of AIOD/PAD and AAA. The major findings reported in this article are 1) the association of both the *SEPP1 rs7579A* allele, and elevated concentration of SeP with occurrence of the systolic HF in aortic diseases, 2) the association of the *SEPP1 rs3877899A*, *SELENOS rs34713741T*, and *GPX4 rs713041T* alleles with AIOD/PAD, and opposite alleles: *SEPP1 rs3877899G* and *GPX4 rs713041C* with AAA, 3) the cumulative effect of selenoprotein gene variants on the risk of studied aortic diseases, comprising the multiplicative interaction between the *SELENOS rs34713741T* and *GPX4 rs713041T* alleles, affecting the risk and clinical phenotype of AIOD, 4) correlation between SeP levels and BMI in AAA (*r* = -0.575, *P* < .0001). The results are summarized in Fig 3.

These results indicate the partially distinct role of selenoproteins in the pathogenesis of aneurysmal and atherosclerotic ischemic types of arterial diseases, as well as their consequences, such as systolic HF. They confirm and extend our preliminary observation of the opposite role of the *SEPP1 rs3877899A* allele for the development of AAA with (a protective factor) and without (a risk factor) coexisting PAD [29]. The present study also focused on aortic diseases, but examined a larger number of selenoprotein genes in a larger sample of patients and controls (~50% increase). Additionally, it evaluated the cardiac phenotypes and selenoprotein concentration.

The strongest impact of selenoproteins on the pathogenesis of the studied CVDs, was observed in the case of HF. The presence of the *SEPP1 rs7579A* allele was related to an 83% increase in risk. Patients with HF were characterized by increased plasma concentrations of SeP, and SeP levels ≥ 8.5 mg/mL were related to a 3.5-fold increase in the risk of HF. The *SEPP1 rs3877899G–rs7579G* haplotype, which was underrepresented in HF patients, was a negative predictor of SeP levels in this group. Moreover, the Trx levels were elevated in HF; however, this elevation was nonsignificant, so our results could not confirm previous reports on the role of this protein as a biomarker of chronic HF [34]. The observed upregulation of SeP expression may indicate the induction of the antioxidant response to cardiac ischemia related to HF. However, the role of SeP upregulation in CVD is not fully understood. Some previous studies showed that it is favorably expressed in macrophages with anti-atherosclerotic function [35], indicating a protective effect. Others suggested its unfavorable function because, in diabetic condition, this protein can act as an antiangiogenic factor, retarding perfusion recovery in response to ischemia [36]. On the other hand, the link between Se concentration and the development, prevention, and treatment of chronic HF has been well documented. It was demonstrated that long-term multiple micronutrient supplementation containing Se can improve left ventricular volumes, ejection fraction, and the patient’s quality of life (including exercise tolerance); it can also reduce symptoms in elderly patients with chronic HF [4, 5]. Se levels were also linked with the clinical severity of this disease [4]. Because unselective supplementation of Se may be ineffective in HF [37] or even harmful for the CV system [2], while the micronutrient–gene interactions may substantially influence the effectiveness of supplementation [15, 38], the knowledge of genetic variability in selenoprotein genes should be carefully considered in the development of guidelines for Se supplementation in CVDs.

In studied aortic diseases, compared to HF, the impact of a single SNP on the risk was rather modest. In univariate analysis, the presence of the *SELENOS rs34713741T*, *SEPP1 rs3877899A*, or *GPX4 rs713041T* allele was related to a 30–61% increase in AIOD/PAD risk in the recessive or dominant model, while carriers of the *SEPP1 rs3877899A* allele had a 32% reduced risk of AAA (in the absence of PAD). In contrast, relatively strong cumulative two-locus effects of selenoprotein SNPs, but not the *SOD2 rs4880* SNP, on the AIOD/PAD/AAA risk were observed. Both, protective and adverse effects of these SNPs were identified, among which specific and nonspecific to the disease occurred. In general, these specific two-locus effects resulted in 50% reduced susceptibility to aneurysm development, whereas in the case of AIOD, they caused from 1.6 to 6 times greater susceptibility. The opposite effects of selenoprotein gene variants on the pathogenesis of AAA and AIOD revealed the complex nature of their influence on CVDs.

Considering the two-locus effects, the most important one resulted in the interaction between the *GPX4* and *SELENOS* SNPs. A 5.3-fold increase in AIOD risk was found for homozygous carriers of the *rs713041T* and *rs34713741T* alleles, compared to the risk in homozygous and heterozygous carriers of the *rs713041C* and *rs34713741C* alleles. The observed impact of the *rs713041TT/rs34713741TT* genotype was 4.4- times greater than estimated from separate effects of the single SNP, which indicated a multiplicative interaction. The association of the *rs713041TT/rs34713741TT* genotype with AIOD remained significant after adjustment for diabetes, obesity and the low HDLC level, and becomes stronger with age. Patients with AIOD carrying this genotype were characterized by older age and higher frequency of diabetes and obesity, factors that are known to cause depression of the antioxidant system. In addition, the same interaction between the *GPX4* and *SELENOS* SNPs was observed in the analysis that compares the frequency of genotypes between AIOD and AAA, which confirmed the substantial differences in genetic predisposition to atherosclerosis and aneurysm.

The observed two-locus effects were relatively strong, partly independent of the influence of known CV risk factors, and each of them explained some portion of the overall susceptibility as well as the differences in the genetic background between AIOD and AAA. For example, the *GPX4 rs713041TT/SELENOS rs34713741TT* genotype was observed in 4% of the AIOD patients and only 0.8% of the AAA patients without concomitant PAD. On the other hand, 9% of the AIOD subjects were carriers of the *GPX4 rs713041CC/SEPP1 rs3877899GA+AA* genotypes, which decrease the AIOD risk by 64%. Similar effects of two-locus interactions between selenoprotein genes were previously observed in relation to the risk of colorectal, breast, and prostate cancer [18–20]. According to BioGrid, IntAct, and STRING databases, there are no known protein-protein interactions between intracellular selenoproteins; therefore, the observed two-locus effects seem to be a consequence of functional relationships.

The effects of minor frequency alleles on the susceptibility to CVD, were in this study compatible between HF and AIOD/PAD (factors increasing the risk), which implies the common features of pathogenesis. In fact, the atherosclerotic ischemic heart disease is the major cause of the chronic HF at an older age. Also due to the similarity in pathogenesis, HF was more frequently found in AIOD, than in AAA, despite the fact that patients with aneurysms were older and, therefore, more prone to development of the left ventricular dysfunction. In the case of aortic diseases, the influence of known CV risk factors is the most likely factor that contributes to the difference in the impact of minor frequency alleles on the risk. Diabetes and cigarette smoking, which are considered to be more potent risk factors for atherosclerosis than AAA [39], interact with Se metabolism and utilization [8, 40] as well as selenoprotein concentration and activity [24].

SNPs that were associated with AIOD/PAD and HF are located in genes that encode proteins involved in the protection of cells from damage caused by oxidative stress and inflammatory conditions related to tissue hypoxia. SeP, located mainly in plasma, acts as a major extracellular antioxidant [41], while SelS and PHGPx are important intracellular antioxidants [42, 43]. PHGPx is an enzyme involved in removing oxidative modifications from lipids, the accumulation of which in the arterial wall is a major contributor to the pathogenesis of atherosclerosis [44]. There is an increasing number of studies showing the role of selenoprotein gene variants in CVD. The *SELENOS* SNPs in the promoter region were linked to the development of diabetes [24], ischemic stroke [21, 22], coronary heart disease [22, 23], as well as carotid and coronary atherosclerosis [23]. The *SEPP1 rs3877899* and *rs7579* SNPs have been associated with metabolic phenotypes related to diabetes [28], while different *GPX4* variants were linked with the concentration of inflammatory biomarkers [27], glutathione peroxidase activity [45], lipid metabolism [46], as well as obesity [45], cerebral stroke [25], and endothelial dysfunction [26]. Also in this study, some of the above-mentioned variants were correlated with selenoprotein concentration; however, these relationships did not pertain to the whole studied population, which might be attributed to the influence of confounding factors related to both the diseases and environmental exposure. Worth noticing that the role of minor frequency alleles in AIOD/PAD and HF was consistent with the idea of selective pressure against deleterious non‐synonymous SNPs [47].

Chronic HF and lower extremity ischemia due to PAD significantly increase the risk of perioperative mortality in open and endovascular surgery for repairing AAA [48, 49]. HF is also a disqualifying medical condition for open surgical implantation of aortic prostheses in both AAA and AIOD [50, 51]. Even after successful surgical treatment, the risk of CV death and recurrent hospital admissions is significantly higher in patients with concomitant HF. Thus, the selenoprotein variants and SeP levels may be promising markers for the identification of this high-risk group, which will enable the provision of special care to these patients, including nutritional strategies to restore redox balance.

In the extension of the results of our previous work, a negative correlation between the concentration of SeP and BMI values in AAA has been demonstrated. This relationship may explain the previously observed impact of the *SEPP1 rs3877899G-rs7579G* haplotype on the risk of AAA in subjects with BMI ≥ 25. Excessive weight is related to immune system activation, chronic inflammation, and eventually faster consumption of antioxidants. Ultimately, reduced antioxidant reserve efficiency in overweight and obese individuals might result in revealing of the pathogenic potential of *SEPP1* SNPs.

There are some limitations in the present study. Firstly, it was designed as a hospital-based study, so the possibility of selection bias could not be ruled out. Secondly, the *SELENOS rs34713741T* variant deviates from HWE in the studied controls (significant at *P* = .028). In an independent analysis, we observed that *SELENOS rs34713741T* allele in recessive model was strongly related to the development of PAD in diabetic subjects (data not shown), which most probably contributed to the random (without prior knowledge of the genotype) exclusion of subjects with this genotype from the control group, because of the presence of PAD symptoms (assessed on the basis of ABI). This derogation did not affect the results and conclusions, because the results of a case-control analysis were confirmed in the analysis of differences in genotype distribution between AIOD/PAD and AAA patients. The third limitation is that the results concerning the concentration of selenoproteins were obtained on a limited group of patients and controls, therefore they should be confirmed in a larger study population. Finally, this study did not assess the level of SeP in tissues and the concentration of Se in both tissues and plasma. SeP is also expressed in many tissues [52], therefore the concentration of this protein in the patient's heart may differ from those in plasma. Moreover, measurement of the Se content in hair or nails might be a better biomarker of Se/selenoprotein status, compared to the blood concentration of Se or SeP, as it offers a long-term marker.

In conclusion, our results provide evidence that increased plasma concentration of SeP and the presence of the *SEPP1 rs7579A* allele are markers for HF development/risk in aortic diseases. The selenoprotein variants *SELENOS rs34713741T*, *GPX4 rs713041T*, and *SEPP1 rs3877899A* constitute risk factors for PAD. Simultaneously, the *GPX4 rs713041T* and *SEPP1 rs3877899A* alleles are preventing factors for the development of AAA, which confirms the substantial differences in genetic predisposition to atherosclerosis and aneurysm. Excessive weight through reduced antioxidant reserve efficiency might result in revealing of the pathogenic potential of selenoprotein SNPs in AAA. Clinical studies are needed to describe the role of these variants in larger cohorts and to define the relationships between them and the status of selenoproteins and Se. The concentration of SeP and Se in both plasma and tissues of patients with HF should also be investigated in further studies to generalize our findings regarding the pathomechanism of HF development and to prepare risk scores and recommendations.

## Supporting information

S1 Table Clinical characteristics of patients with abdominal aortic aneurysm (AAA) stratified according to peripheral arterial disease (PAD) coexistence. (DOCX)

S2 Table TaqMan SNP Genotyping Assays and the Hardy-Weinberg equilibrium analysis. (DOCX)

S3 Table Distribution of genotypes and alleles of the studied polymorphisms in patients with abdominal aortic aneurysm (AAA) stratified according to peripheral arterial disease (PAD) coexistence, patients with aortoiliac occlusive disease (AIOD), and controls. (DOCX)

S4 Table Distribution of studied polymorphisms in patients with abdominal aortic aneurysm (AAA) and aortoiliac occlusive disease (AIOD) stratified according to cardiac phenotypes and presence of peripheral arterial disease (PAD). (DOCX)

S5 Table Cumulative effects of polymorphic alleles. Odds ratios (ORs) are presented with reference to all other genotype combinations.
(DOCX)

S6 Table Multivariate analysis of associations of the *SEPP1 rs34713741G>A*, *SELENOS rs34713741C>T*, *TXNRD2 rs9605031C>T*, and *GPX4 rs713041C>T* polymorphisms with abdominal aortic aneurysm (AAA) and aortoiliac occlusive disease (AIOD). (DOCX)

S7 Table Genotype data set. (XLSX)
