# Supplementary material for: Associations and interactions between variants in selenoprotein genes, selenoprotein levels and the development of abdominal aortic aneurysm, peripheral arterial disease, and heart failure
Source: PLoS One. 2018 Sep 6;13(9):e0203350. doi: 10.1371/journal.pone.0203350 (PMC6126836; doi:10.1371/journal.pone.0203350)
Supplement: S1 Table — (DOCX) [file pone.0203350.s001.docx]

| **S1 Table**│ TaqMan SNP Genotyping Assays and the Hardy-Weinberg equilibrium analysis. | | | | | | |  | | | | | |
| --- | --- | --- | --- | --- | --- | --- | --- | --- | --- | --- | --- | --- |
| SNP | TaqMan SNP Genotyping Assay | SNP localization | MAF_POP_ | Obs.  % Het | Pred.  % Het | *P_HWE_* | MAF_CON_ | *P_HWE_* | MAF_AAA_ | *P_HWE_* | MAF_AIOD_ | *P_HWE_* |
| *SEPP1 rs3877899* | C___2841533_10 | Intergenic | 0.242 | 37.4 | 36.6 | *0.415* | 0.259 | *0.598* | 0.227 | *0.237* | 0.239 | *0.741* |
| *SEPP1 rs7579* | C___8806056_10 | 3’UTR | 0.290 | 41.5 | 41.2 | *0.772* | 0.282 | *0.062* | 0.296 | *0.062* | 0.291 | *0.640* |
| *SELENOS rs34713741* | C___3091980_10 | Intron | 0.326 | 44.1 | 43.9 | *0.837* | 0.322 | *0.028* | 0.320 | *0.452* | 0.339 | *0.255* |
| *TXNRD1* *rs35009941* | C___25592684_10 | Intron | 0.001 | 0.3 | 0.3 | *0.959* | 0.001 | *0.983* | 0.002 | *0.983* | 0.001 | *0.980* |
| *TXNRD2* *rs9605031* | C___30606022_10 | Intron | 0.279 | 39.3 | 40.2 | *0.393* | 0.290 | *0.268* | 0.257 | *0.655* | 0.295 | *0.443* |
| *GPX4* *rs713041* | C___2561693_20 | Intergenic | 0.413 | 48.6 | 48.5 | *0.945* | 0.406 | *0.310* | 0.402 | *0.253* | 0.438 | *0.909* |
| *SOD2 rs4880* | C___8709053_10 | 5’UTR | 0.475 | 48.7 | 49.9 | *0.376* | 0.477 | *0.902* | 0.477 | *0.379* | 0.470 | *0.596* |
| Abbreviations: *3’UTR*, 3′ untranslated region; *5’UTR*, 5′ untranslated region; *AAA,* patients with abdominal aortic aneurysm; *AIOD,* patients with aortoiliac occlusive disease*; CON,* control subjects*; % Het,* percentage of heterozygotes; *MAF,* minor allele frequency*; Obs*., observed; *P_HWE_ –* Hardy–Weinberg equilibrium *P* values*; POP,* the whole studied population*; Pred*., predicted; *SNP*, single nucleotide polymorphism | | | | | | | | | | | | |
