# Supplementary material for: Associations and interactions between variants in selenoprotein genes, selenoprotein levels and the development of abdominal aortic aneurysm, peripheral arterial disease, and heart failure
Source: PLoS One. 2018 Sep 6;13(9):e0203350. doi: 10.1371/journal.pone.0203350 (PMC6126836; doi:10.1371/journal.pone.0203350)
Supplement: S2 Table — (DOCX) [file pone.0203350.s002.docx]

| S2 Table│ Clinical characteristics of patients with abdominal aortic aneurysm (AAA) stratified according to peripheral arterial disease (PAD) coexistence. | | | |
| --- | --- | --- | --- |
| Variable | AAA  without PAD  N=259 | AAA  with PAD  N=263 | *P* value for comparison of groups |
|  |  |  |  |
| Median age, years | 70 (62, 75) | 68 (62, 74) | .158 |
| Age range, years | 40 - 89 | 46 - 94 |  |
| Male sex | 220 (84.9) | 228 (86.7) | .567 |
| Smoking | 209 (80.7) | 219 (83.2) | .440 |
| Arterial hypertension | 204 (80.7) | 180 (68.4) | .008 |
| Diabetes | 39 (15.1) | 44 (16.7) | .882 |
| Obesity | 52 (20.1) | 42 (15.9) | .222 |
| Lipid and lipoprotein profile, mmol/L | | | |
| TC | 5.18 (4.26, 6.04) | 5.17 (4.32, 6.07) | .637 |
| HDLC | 1.13 (0.93, 1.36) | 1.11 (0.92, 1.36) | .762 |
| LDLC | 3.10 (2.30, 4.00) | 3.10 (2.39, 4.00) | .670 |
| TG | 1.36 (1.02, 2.02) | 1.53 (1.12, 2.07) | .108 |
| Comorbidities | | | |
| Coronary artery disease | 125 (47.9) | 145 (58.5) | .116 |
| Myocardial infarction | 85 (32.4) | 84 (31.9) | .830 |
| Systolic heart failure | 16 (6.2) | 13 (4.9) | .538 |
| Peripheral arterial disease | 0 (0.0) | 263 (100.0) | Not applicable |
| Aortic diameter, mm | 60 (54, 70) | 60 (54, 71) | .912 |
| Variables are expressed as median (interquartile range) or as percentages.  *HDLC*, high-density lipoprotein cholesterol; *LDLC*, low-density lipoprotein cholesterol; *TC*, total plasma cholesterol; *TG*, triglyceride. | | | |
