# Supplementary material for: Associations and interactions between variants in selenoprotein genes, selenoprotein levels and the development of abdominal aortic aneurysm, peripheral arterial disease, and heart failure
Source: PLoS One. 2018 Sep 6;13(9):e0203350. doi: 10.1371/journal.pone.0203350 (PMC6126836; doi:10.1371/journal.pone.0203350)
Supplement: S3 Table — (DOCX) [file pone.0203350.s003.docx]

| **S3** **Table** │ Distribution of genotypes and alleles of the studied polymorphisms in patients with abdominal aortic aneurysm (AAA) stratified according to peripheral arterial disease (PAD) coexistence, patients with aortoiliac occlusive disease (AIOD), and controls. | | | | | | | | | | | | |
| --- | --- | --- | --- | --- | --- | --- | --- | --- | --- | --- | --- | --- |
| Genotype | Controls  (I)  N=543 | | AAA | | | | | | AIOD  (V)  N=400 | | Statistical analysis  Compared groups,  model of genetic association: *OR (95%CI), P* | |
|  |  |  | All  (II)  N=564 | | Without PAD  (III)  N=259 | | With PAD  (IV)  N=263 | |  |  |  |  |
| *SEPP1 rs3877899G>A* | | | | | | | | | | | | |
| *GG* | 296 (54.5) | | 331 (58.9) | | 165 (63.7) | | 140 (53.6) | | 233 (58.3) | | *III vs I*, dominant:  0.68 (0.50-0.93), .014  *IV vs III,* dominant:  1.52 (1.07-2.16), .020 | |
| *GA* | 213 (39.2) | | 207 (36.8) | | 84 (32.4) | | 108 (41.4) | | 143 (35.8) | |  |  |
| *AA* | 34 (6.3) | | 24 (4.3) | | 10 (3.9) | | 13 (5.0) | | 24 (6.0) | |  |  |
| *MAF* | 0.259 | | 0.227 | | 0.201 | | 0.257 | | 0.239 | |  |  |
| *SEPP1 rs7579 G>A* | | | | | | | | | | | | |
| *GG* | 288 (53.1) | | 270 (48.0) | | 122 (47.3) | | 127 (48.7) | | 199 (49.9) | |  | |
| *GA* | 202 (37.3) | | 253 (44.9) | | 115 (44.6) | | 119 (44.8) | | 169 (42.3) | |  |  |
| *AA* | 52 (9.6) | | 40 (7.1) | | 21 (8.1) | | 17 (6.5) | | 32 (8.0) | |  |  |
| *MAF* | 0.282 | | 0.296 | | 0.304 | | 0.291 | | 0.291 | |  |  |
| *SELENOS rs34713741C>T* | | | | | | | | | | | | |
| *CC* | 238 (43.9) | | 265 (47.0) | | 129 (49.8) | | 123 (46.8) | | 180 (45.0) | | *V vs I,* recessive.:  1.61 (1.06-2.47), .026 | |
| *CT* | 259 (47.8) | | 237 (42.0) | | 101 (39.0) | | 112 (42.6) | | 169 (42.3) | |  |  |
| *TT* | 45 (8.3) | | 62 (11.0) | | 29 (11.2) | | 28 (10.7) | | 51 (12.8) | |  |  |
| *MAF* | 0.322 | | 0.320 | | 0.307 | | 0.319 | | 0.339 | |  |  |
| *TXNRD1 rs35009941C>G* | | | | | | | | | | | | |
| *CC* | 541 (99.8) | | 562 (100.0) | | 259 (100.0) | | 261 (99.2) | | 397 (99.8) | |  | |
| *CG* | 1 (0.2) | | 2 (0.35) | | 0 (0.0) | | 2 (0.8) | | 1 (0.3) | |  |  |
| *MAF* | 0.001 | | 0.002 | | 0.000 | | 0.004 | | 0.001 | |  |  |
| *TXNRD2 rs9605031C>T* | | | | | | | | | | | | |
| *CC* | 279 (51.4) | | 309 (54.8) | | 134 (51.7) | | 148 (56.3) | | 202 (50.1) | |  | |
| *CT* | 213 (39.2) | | 220 (39.0) | | 110 (42.5) | | 95 (36.1) | | 160 (40.0) | |  |  |
| *TT* | | 51 (9.4) | | 35 (6.2) | | 15 (5.8) | | 20 (7.6) | | 38 (9.5) | |  |
| *MAF* | | 0.290 | | 0.257 | | 0.270 | | 0.257 | | 0.295 | |  |
| *GPX4 rs713041C>T* | | | | | | | | | | | | |
| *CC* | | 197 (36.3) | | 195 (34.6) | | 101 (39.2) | | 76 (28.9) | | 126 (31.5) | | *IV vs I,* dominant:  1.41 (1.02-1.93), .036;  *IV vs III*, dominant:  1.58 (1.10-2.28), .014;  *V vs III,* dominant:  1.40 (1.0-1.94), .044 |
| *CT* | | 250 (46.1) | | 283 (50.3) | | 119 (46.1) | | 148 (56.3) | | 198 (45.5) | |  |
| *TT* | | 95 (17.5) | | 85 (15.1) | | 38 (14.7) | | 39 (14.8) | | 76 (19.0) | |  |
| *MAF* | | 0.406 | | 0.402 | | 0.378 | | 0.430 | | 0.438 | |  |
| *SOD2 rs4880C>T* | | | | | | | | | | | | |
| *CC* | | 149 (27.5) | | 159 (28.3) | | 72 (27.9) | | 76 (29.0) | | 115 (28.8) | |  |
| *CT* | | 269 (49.6) | | 270 (48.0) | | 119 (46.2) | | 132 (50.4) | | 194 (48.5) | |  |
| *TT* | | 124 (22.9) | | 133 (23.7) | | 67 (26.0) | | 54 (20.6) | | 91 (22.8) | |  |
| *MAF* | | 0.477 | | 0.477 | | 0.490 | | 0.458 | | 0.470 | |  |
| *GPX4,* the glutathione peroxidase 4 gene*; MAF, minor allele frequency;*  *SELENOS, the selenoprotein S gene; SEPP1, the selenoprotein P gene; SOD2, the manganese superoxide dismutase gene; TXNRD1; the thioredoxin reductase 1 gene; TXNRD2; the thioredoxin reductase 2 gene* | | | | | | | | | | | | |
