# Supplementary material for: Associations and interactions between variants in selenoprotein genes, selenoprotein levels and the development of abdominal aortic aneurysm, peripheral arterial disease, and heart failure
Source: PLoS One. 2018 Sep 6;13(9):e0203350. doi: 10.1371/journal.pone.0203350 (PMC6126836; doi:10.1371/journal.pone.0203350)
Supplement: S4 Table — (DOCX) [file pone.0203350.s004.docx]

| **S4** **Table** │ Distribution of studied polymorphisms in patients with abdominal aortic aneurysm (AAA) and aortoiliac occlusive disease (AIOD) stratified according to cardiac phenotypes and presence of peripheral arterial disease (PAD). | | | | | | | | |
| --- | --- | --- | --- | --- | --- | --- | --- | --- |
| SNP | Coronary artery disease | | Myocardial infarction | | Systolic heart failure | | Peripheral arterial disease | |
|  | absent (I)  N=514 | present (II)  N=450 | absent (III)  N=684 | present (IV)  N=280 | absent (V)  N=883 | present (VI)  N=81 | absent (VII)  N=259 | present (VIII)  N=661 |
| *SEPP1 rs3877899- rs7579* haplotypes | | | | | | | | |
| *G - G* |  | |  | |  | |  |  |
| *0* | 137 (26.8) | 125 (27.8) | 185 (27.1) | 77 (27.6) | 232 (26.4) | 30 (37.0) | 68 (26.4) | 184 (27.9) |
| *1* | 255 (49.8) | 231 (51.4) | 343 (50.3) | 143 (51.3) | 447 (50.8) | 39 (48.1) | 124 (48.1) | 337 (51.1) |
| *2* | 120 (23.4) | 93 (20.7) | 154 (22.6) | 59 (21.1) | 201 (22.8) | 12 (14.8) | 66 (25.6) | 138 (20.9) |
| *HF* | 0.483 | 0.464 | 0.477 | 0.468 | **0.482^a^** | **0.389^a^** | 0.496 | 0.465 |
| *G - A* |  | |  | |  | |  |  |
| *0* | 258 (50.4) | 210 (46.8) | 337 (49.4) | 131 (47.0) | 437 (49.7) | 31 (38.3) | 121 (46.9) | 325 (49.3) |
| *1* | 216 (42.2) | 205 (45.7) | 293 (43.0) | 128 (45.9) | 376 (42.7) | 45 (55.6) | 117 (45.3) | 285 (43.2) |
| *2* | 38 (7.4) | 34 (7.6) | 52 (7.6) | 20 (7.2) | 67 (7.6) | 5 (6.2) | 20 (7.8) | 49 (7.4) |
| *HF* | 0.285 | 0.304 | 0.291 | 0.301 | **0.290^b^** | **0.340^b^** | 0.304 | 0.291 |
| *A - G* |  | |  | |  | |  |  |
| *0* | 300 (58.6) | 264 (58.8) | 400 (58.7) | 164 (58.8) | 522 (59.3) | 42 (51.9) | 165 (64.0) | 373 (56.6) |
| *1* | 187 (36.5) | 162 (36.1) | 248 (36.4) | 101 (36.2) | 315 (35.8) | 34 (42.0) | 83 (32.2) | 250 (37.9) |
| *2* | 25 (4.9) | 23 (5.1) | 34 (5.0) | 14 (5.0) | 43 (4.9) | 5 (6.2) | **10 (3.9)^c^** | **36 (5.5)^c^** |
| *HF* | 0.231 | 0.232 | 0.232 | 0.231 | 0.228 | 0.272 | 0.200 | 0.244 |
| *SELENOS rs34713741* | | |  | |  | |  |  |
| *CC* | 235 (45.7) | 210 (46.7) | 307 (44.9) | 138 (49.3) | 404 (45.8) | 41 (50.6) | 129 (49.8) | 303 (45.7) |
| *CT* | 214 (41.6) | 192 (42.7) | 292 (42.7) | 114 (40.7) | 374 (42.4) | 32 (39.5) | 101 (39.0) | 281 (42.3) |
| *TT* | 65 (12.6) | 48 (10.7) | 85 (12.4) | 28 (10.0) | 105 (11.9) | 8 (9.9) | 29 (11.2) | 79 (11.9) |
| *MAF* | 0.335 | 0.320 | 0.338 | 0.304 | 0.331 | 0.296 | 0.307 | **0.331^d^** |
| *TXNRD2 rs9605031* | | |  | |  | |  |  |
| *CC* | 273 (53.3) | 238 (52.9) | 360 (52.6) | 151 (53.9) | 467 (52.9) | 44 (54.3) | 134 (51.7) | 350 (52.8) |
| *CT* | 204 (39.8) | 174 (38.7) | 273 (39.9) | 107 (38.2) | 350 (39.6) | 30 (37.0) | 110 (42.5) | 255 (38.4) |
| *TT* | 35 (6.8) | 38 (8.4) | 51 (7.5) | 22 (7.9) | 66 (7.5) | 7 (8.6) | 15 (5.8) | 58 (8.7) |
| *MAF* | 0.268 | 0.278 | 0.274 | 0.270 | 0.273 | 0.272 | 0.270 | 0.280 |
| *GPX4 rs713041* | | |  | |  | |  |  |
| *CC* | 171 (33.3) | 150 (33.4) | 233 (34.1) | 88 (31.5) | 296 (33.6) | 25 (30.9) | 101 (39.2) | 202 (30.5) |
| *CT* | 256 (49.8) | 226 (50.3) | 341 (49.9) | 141 (50.5) | 438 (49.7) | 44 (54.3) | 119 (46.1) | 346 (52.2) |
| *TT* | 87 (16.9) | 73 (16.3) | 110 (16.1) | 50 (17.9) | 148 (16.8) | 12 (14.8) | **38 (14.7)^e^** | **115 (17.3)^e, f^** |
| *MAF* | 0.418 | 0.414 | 0.410 | 0.432 | 0.416 | 0.420 | 0.378 | 0.434 |
| *SOD2 rs4880* | | |  | |  | |  |  |
| *CC* | 147 (28.7) | 127 (28.3) | 194 (28.4) | 80 (28.7) | 257 (29.2) | 17 (21.0) | 72 (27.9) | 191 (28.8) |
| *CT* | 246 (48.0) | 218 (48.6) | 333 (48.8) | 131 (47.0) | 417 (47.3) | 47 (58.0) | 119 (46.2) | 326 (49.2) |
| *TT* | 120 (23.4) | 104 (23.2) | 156 (22.8) | 68 (24.4) | 207 (23.5) | 17 (21.0) | 67 (26.0) | 145 (21.9) |
| *MAF* | 0.474 | 0.474 | 0.472 | 0.478 | 0.472 | 0.500 | 0.490 | 0.465 |
| *HF*, haplotype frequency; *MAF*, minor allele frequency  ^a-d^ Major associations of alleles and haplotypes with the occurrence of concomitant diseases in AAA and AIOD  Compared groups, allele or haplotype, model tested: OR (95%CI)*, P*  *a* – systolic heart failure, *VI vs V*, *SEPP1 G-G* haplotype carriers: OR= 0.61 (0.38 to 0.98), *P*=.039;  *b* - systolic heart failure, *VI vs V*, *SEPP1 G-G* haplotype carriers: OR= 1.59 (1.0-2.54), *P*<0.050; (VI vs controls*: OR= 1.83 (1.13-2.95), *P*=.0125)  *c* - peripheral arterial disease, *VIII vs VII*, *SEPP1 A-G* haplotype carriers: OR=1.36 (1.01-1.83), *P*=.04  *d* - peripheral arterial disease, *VIII vs* controls*: *SELENOS rs34713741T* allele recessive model: OR=1.49 (1.02-2.20), *P*=0.040  *e* - peripheral arterial disease, *VIII vs VII*: *GPX4 rs713041T* allele*,* dominant model: OR= 1.47 (1.10-1.98), *P*=.012  *f -* peripheral arterial disease, *VIII* vs controls*: OR=1.30 (1.02-1.65), *P*=.031.  *-genotype frequencies in the control group are shown in Table S3. | | | | | | | | |
