# Supplementary material for: Associations and interactions between variants in selenoprotein genes, selenoprotein levels and the development of abdominal aortic aneurysm, peripheral arterial disease, and heart failure
Source: PLoS One. 2018 Sep 6;13(9):e0203350. doi: 10.1371/journal.pone.0203350 (PMC6126836; doi:10.1371/journal.pone.0203350)
Supplement: S5 Table — Odds ratios (ORs) are presented with reference to all other genotype combinations. (DOCX) [file pone.0203350.s005.docx]

| S5 Table │ Cumulative effects of polymorphic alleles. Odds ratios (ORs) are presented with reference to all other genotype combinations. | | | | | |
| --- | --- | --- | --- | --- | --- |
| Studied genetic variants | | Genotype  combination | Cases/  Reference | OR (95% CI) | *P* value |
| AAA (N=564) *vs* Controls (N=543) | | | | | |
| *GPX4* | *SEPP1 SNP1* | *CC / GA+AA* | 73/102 | 0.65 (0.47 - 0.89) | .008* |
| *TXNRD2* | *SEPP1 SNP1* | *TT / GG* | 17/31 | 0.52 (0.28 - 0.94) | .029 |
| *TXNRD2* | *SELENOS^#^* | *TT / CC+CT* | 30/51 | 0.54 (0.34 - 0.86) | .009* |
| AIOD (N=400) *vs* Controls (N=543) | | | | | |
| *GPX4* | *SEPP1 SNP1* | *CC / GA+AA* | 35/102 | 0.41 (0.28 - 0.62) | < .0001* |
| *GPX4* | *SELENOS^#^* | *TT / TT* | 15/4 | 5.23 (1.72 - 15.9) | .001* |
| *GPX4* | *TXNRD2* | *CT+TT / CT+TT* | 347/436 | 1.59 (1.11 - 2.28) | .011* |
| *TXNRD2* | *SELENOS^#^* | *TT / TT* | 8/0 | 23.5 (1.13 – 40.6) | .001* |
| *SELENOS* | *SEPP1 SNP2* | *TT / GG+GA* | 49/37 | 1.90 (1.22 - 3.00) | .004* |
| AIOD (N=400) *vs* AAA (N=564) | | | | | |
| *GPX4* | *SELENOS* | *TT / TT* | 15/7 | 3.10 (1.25 - 7.68) | .010* |
| *GPX4* | *SELENOS* | *TT / CT+TT* | 48/40 | 1.72 (1.01 – 2.93) | .046 |
| *GPX4* | *TXNRD2* | *TT / CC* | 42/44 | 1.91 (1.12 - 3.26) | .017* |
| *GPX4* | *TXNRD2* | *CT+TT / TT* | 22/18 | 2.44 (1.21 - 4.95) | .012* |
| *GPX4* | *SOD2* | *TT / CT* | 41/35 | 1.86 (1.00 – 3.47) | <.050 |
| *TXNRD2* | *SEPP1 SNP1* | *TT / GG* | 25/17 | 2.14 (1.14 - 4.01) | .016* |
| AIOD (N=400) *vs* AAA without PAD (N=259) | | | | | |
| *GPX4* | *SELENOS* | *TT / TT* | 2/7 | 6.00 (1.31 – 27.5) | .011* |
| *GPX4* | *SELENOS* | *TT / CT+TT* | 18/40 | 2.13 (1.10 - 4.13) | .024* |
| *GPX4* | *TXNRD2* | *TT / CC* | 16/44 | 2.58 (1.29 - 5.14) | .007* |
| *GPX4* | *TXNRD2* | *CT+TT / TT* | 7/18 | 3.08 (1.21 - 7.84) | .015* |
| *GPX4* | *SOD2* | *TT / CT* | 12/35 | 2.41 (1.05 - 5.53) | .035* |
| *TXNRD2* | *SEPP1 SNP1* | *TT / GG* | 6/17 | 3.05 (1.20 - 7.78) | .015* |
| AAA+AIOD (N=961) *vs* Controls (N=543) | | | | | |
| *GPX4* | *SEPP1 SNP1* | *CC / GG+GA* | 108/102 | 0.55 (0.41 - 0.73) | < .0001* |
| *TXNRD2* | *SELENOS* | *TT / TT* | 13/0 | 15.4 (0.91 - 256.9) | .006* |
| The *SEPP1 rs3877899 (SEPP1 SNP1),* *SEPP1 rs7579 (SEPP1 SNP2), SELENOS rs34713741, TXNRD2 rs9605031,* *GPX4 rs713041,* and *SOD2* *rs4880* polymorphisms were analyzed. *P* values (crude) for comparison of studied and reference groups are shown. *P* values significant after Bonferroni correction for multiple comparisons are indicated with an asterisk (*). | | | | | |
