# Supplementary material for: Associations and interactions between variants in selenoprotein genes, selenoprotein levels and the development of abdominal aortic aneurysm, peripheral arterial disease, and heart failure
Source: PLoS One. 2018 Sep 6;13(9):e0203350. doi: 10.1371/journal.pone.0203350 (PMC6126836; doi:10.1371/journal.pone.0203350)
Supplement: S6 Table — (DOCX) [file pone.0203350.s006.docx]

| **S6 Table** **│** Multivariate analysis of associations of the *SEPP1 rs34713741G>A, SELENOS rs34713741C>T,* *TXNRD2* *rs9605031C>T,* and *GPX4 rs713041C>T* polymorphisms with abdominal aortic aneurysm (AAA) and aortoiliac occlusive disease (AIOD). | | |
| --- | --- | --- |
| Genotype | OR*_Adjusted_* (95%CL), *P* | Cardiovascular risk factors in studied model |
| **Effects of single allele** | | |
| 1. AIOD vs Controls^a^ | | |
| - *SELENOS TT* | 1.91 (1.17- 3.10), **.009** | T2DM, BMI>30, low HDLC |
| - Dose of *GPX4* *T* allele | 1.19 (0.96-1.48), .107 |  |
| 2. AAA PAD vs Controls | | |
| - *GPX4* *CT+TT* | 1.74 (1.18- 2.55), **.005** | low HDLC, age, sex |
| **Effects of combination of genotypes**^b^ | | |
| 3. AIOD vs Controls | | |
| - *SELENOS TT/ GPX4* *TT* | 5.43 (1.68-17.5), **.005** | T2DM, BMI>30, low HDLC |
| - *SEPP1 GA+AA/ GPX4* *CC* | 0.39 (0.24-0.62), **<.0001** |  |
| - *GPX4* *CT+TT/ TXNRD2 CT+TT* | 1.74 (1.15- 2.65), **.009** |  |
| - *SELENOS TT/* *SEPP1 GG+GA* | 2.27 (1.36- 3.79), **.002** |  |
| 4. AIOD vs AAA | | |
| - *SELENOS TT/ GPX4* *TT* | 4.85 (1.79-13.1), **.002** | T2DM, BMI>30, age, sex |
| 5. AAA vs Controls | | |
| - *SEPP1 GA+AA/ GPX4* *CC* | 0.61 (0.41-0.91), **.020** | low HDLC, age, sex |
| 6. AAA+AIOD vs Controls | | |
| - *SEPP1 GA+AA/ GPX4* *CC* | 0.46 (0.32-0.67), **<.0001** | BMI>30, low HDLC, age, sex |
| ^a^-both, the *SELENOS* and *GPX4* genotypes were analyzed in one model  ^b^-each combination of genotypes was studied in separate model  *BMI*, body mass index; *HDLC,* high-density lipoprotein cholesterol; *low HDLC*, <1.0 mmol/L for men and <1.2 mmol/L for women; *T2DM*, type 2 diabetes | | |
